# Information mismatch in PHH3-assisted mitosis annotation leads to interpretation shifts in H&E slide analysis

**DOI:** 10.1038/s41598-024-77244-6

**Published:** 2024-11-01

**Authors:** Jonathan Ganz, Christian Marzahl, Jonas Ammeling, Emely Rosbach, Barbara Richter, Chloé Puget, Daniela Denk, Elena A. Demeter, Flaviu A. Tăbăran, Gabriel Wasinger, Karoline Lipnik, Marco Tecilla, Matthew J. Valentine, Michael J. Dark, Niklas Abele, Pompei Bolfa, Ramona Erber, Robert Klopfleisch, Sophie Merz, Taryn A. Donovan, Samir Jabari, Christof A. Bertram, Katharina Breininger, Marc Aubreville

**Affiliations:** 1 Technische Hochschule Ingolstadt, Ingolstadt, Germany; 2 Gestalt Diagnostics, Spokane, USA; 3 University of Veterinary Medicine, Vienna, Austria; 4 Freie Universität Berlin, Berlin, Germany; 5 Ludwig-Maximilians-Universität München, München, Germany; 6 SeaWorld Yas Island, Abu Dhabi, UAE; 7 Cornell University, Ithaca, USA; 8 University of Agricultural Sciences and Veterinary Medicine of Cluj-Napoca, Cluj-Napoca, Romania; 9 Medical University of Vienna, Vienna, Austria; 10 University of Milan, Milan, Italy; 11 Ross University School of Veterinary Medicine, Basseterre, St. Kitts and Nevis; 12 University of Florida, Gainesville, USA; 13 University Hospital Erlangen, Erlangen, Germany; 14 Universität Regensburg, Regensburg, Germany; 15 IDEXX Laboratories, Kornwestheim, Germany; 16 The Schwarzman Animal Medical Center, New York, USA; 17 Friedrich-Alexander-Universität Erlangen-Nürnberg, Erlangen, Germany; 18 Universität Würzburg, Würzburg, Germany; 19 Flensburg University of Applied Sciences, Flensburg, Germany

**Keywords:** Tumour biomarkers, Computer science

## Abstract

The count of mitotic figures (MFs) observed in hematoxylin and eosin (H&E)-stained slides is an important prognostic marker, as it is a measure for tumor cell proliferation. However, the identification of MFs has a known low inter-rater agreement. In a computer-aided setting, deep learning algorithms can help to mitigate this, but they require large amounts of annotated data for training and validation. Furthermore, label noise introduced during the annotation process may impede the algorithms’ performance. Unlike H&E, where identification of MFs is based mainly on morphological features, the mitosis-specific antibody phospho-histone H3 (PHH3) specifically highlights MFs. Counting MFs on slides stained against PHH3 leads to higher agreement among raters and has therefore recently been used as a ground truth for the annotation of MFs in H&E. However, as PHH3 facilitates the recognition of cells indistinguishable from H&E staining alone, the use of this ground truth could potentially introduce an interpretation shift and even label noise into the H&E-related dataset, impacting model performance. This study analyzes the impact of PHH3-assisted MF annotation on inter-rater reliability and object level agreement through an extensive multi-rater experiment. Subsequently, MF detectors, including a novel dual-stain detector, were evaluated on the resulting datasets to investigate the influence of PHH3-assisted labeling on the models’ performance. We found that the annotators’ object-level agreement significantly increased when using PHH3-assisted labeling (F1: 0.53 to 0.74). However, this enhancement in label consistency did not translate to improved performance for H&E-based detectors, neither during the training phase nor the evaluation phase. Conversely, the dual-stain detector was able to benefit from the higher consistency. This reveals an information mismatch between the H&E and PHH3-stained images as the cause of this effect, which renders PHH3-assisted annotations not well-aligned for use with H&E-based detectors. Based on our findings, we propose an improved PHH3-assisted labeling procedure.

## Introduction

In tumor diagnosis, a crucial step is the examination of tissue samples by pathologists to derive important tumor-related information and aid in determining suitable treatment options. One factor of interest is the proliferation fraction of the respective tumor, which can be assessed through the number of cells undergoing cell division, as observed through mitotic figures (MFs)^1^. The mitotic count (MC), defined as the number of MFs within a standardized area of ten consecutive high-power fields^2^, is known to highly correlate with biological tumor behavior and is part of various tumor grading systems in human^3–6^ as well as veterinary medicine^7–12^. While the identification of MFs is common in routine pathology, it is a task known to have a low inter-rater agreement^13–15^. The availability of slide scanners has enabled the digitization of entire slides into whole slide images (WSIs), which can be analyzed automatically using computer vision methods, and, in particular, deep learning (DL)-based approaches. In the context of MF detection, DL algorithms already demonstrated human-like performance^14^. Nevertheless, those algorithms rely on large amounts of annotated data, and the annotation quality is known to affect the performance of the trained DL model^16,17^. In contrast to the hematoxylin and eosin (H&E) stain, which is the standard stain used in histopathology, staining with the mitosis-specific antibody phosphohistone H3 (PHH3) specifically highlights the cell nucleus during mitosis^18^. The immunohistochemistry (IHC) staining against PHH3 is a validated method with diagnostic and prognostic significance, emphasizing its utility in the assessment of the MC in various tumor types^18–22^. Furthermore, different studies reported that MC determined using PHH3 as sole stain leads to lower variability of the MC among different raters compared with the MC acquired in H&E-stained samples^11,18,21,23,24^. Nevertheless, H&E remains the default stain in pathology due to its widespread adoption and established efficacy for morphological assessment, and therefore, any detectors or diagnostic tools intended for clinical use must be designed to work effectively with this particular stain. Moreover, PHH3 staining is significantly more expensive than H&E staining, rendering it less attractive particularly in cost-sensitive environments such as, for instance, veterinary pathology.

**Fig. 1:**
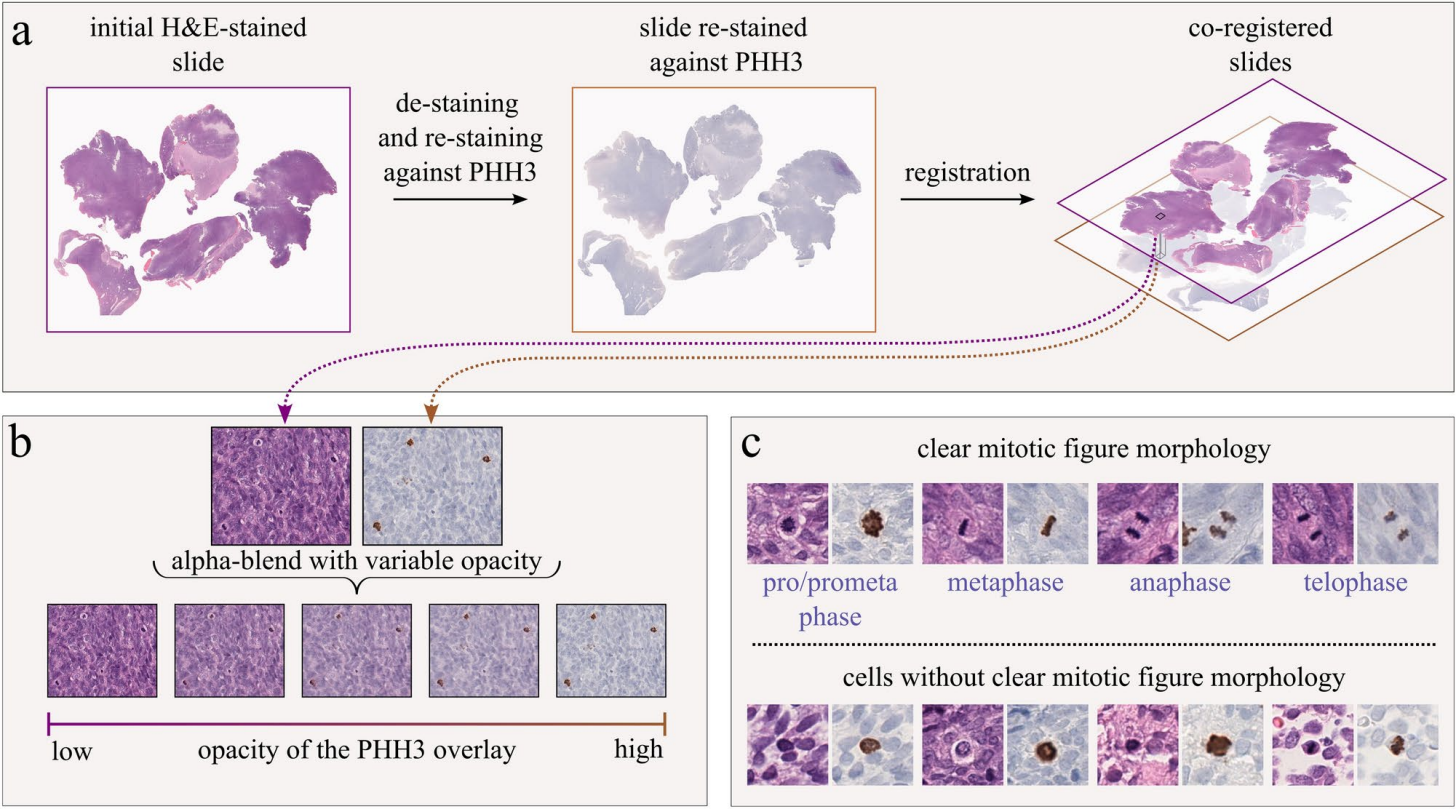
Overview of the PHH3-assisted annotation pipeline. (**a**) In an initial step, the slides were stained with H&E and digitized. Subsequently, the slides were de-stained, immunolabeled with PHH3 and digitized once more. Afterward, the resulting images were registered. (**b**) During the PHH3-assisted labeling process, the experts were able to superimpose the slide stained against PHH3 over its H&E-stained counterpart with varying degrees of opacity, which made it possible to assess the information of both stains simultaneously. (**c**) However, not every cell which exhibits a positive label in the slide stained against PHH3 (brown staining) also possess distinct morphological features in the H&E.

Multiple research groups have explored a workflow that combines both, the superior sensitivity of the PHH3 stain for mitosis identification to improve annotation label quality and the opportunities brought by utilizing machine learning-based detection tools as an assistance for clinicians^25–31^: First, the specimen is stained with one dye (e.g., H&E), and the slide is digitized (see Fig. 1a). After this, the cover-slip is carefully removed and the tissue is de-stained, and subsequently immunohistochemically re-stained (e.g., against PHH3), and digitized again. This workflow yields two digital images that, after a registration, yield cell-exact correspondences, and can be used in tandem to enhance the annotation process, leveraging the complementary information provided by the two stains. This methodology, the application of an IHC stain and the co-registration with H&E-stained images for label generation, has been used before for various applications in digital pathology, e.g., for the segmentation of tissue in colon cancer^32^, prostate cancer^33^, and canine breast cancer^34^, and on a cellular level for cell segmentation in papillary thyroid carcinomas^35^ and melanocytic cells^36^. Given PHH3- and H&E-stained, co-registered images, Tellez et al. proposed to annotate and/or detect on the PHH3-stained image and register these biologically sensible annotations to the corresponding cellular objects within the H&E stain^25^. Ibrahim et al. approached this problem in a different way, by counter-staining slides stained against H&E with PHH3 without prior de-staining, and thus combining both stains within one physical slide^37^. This way, the information of both stains is combined physically in the same image. The authors compared the MCs obtained using their novel staining technique to those derived from classical H&E staining and found that the novel method achieved the highest level of agreement. However, the use of both stains is associated with a significant increase in cost for sample preparation, which reduces the likelihood of an application in routine practice. Hence, while the use of PHH3-stained slides is viable in annotation workflows, for the use in routine pathology diagnostics, it is desirable to use MF detectors that are applicable to solely H&E-stained images. In addition, thresholds related to the MC in grading schemes are based on counting MFs in images stained with H&E^3–12^.

Another option is the concurrent use of both stains in the visual assessment by the pathology expert^14,28,30^, which we denote *PHH3-assisted annotation* in the following. As illustrated in Fig. 1b, during the annotation process, the PHH3 slide is visually overlaid against its H&E-stained counterpart using an alpha-blending technique. The respective expert can then manually adjust the transparency as needed. One of the primary challenges in MF annotation is the high probability of missing MFs during the annotation process^16^. In the PHH3-assisted approach, an expert can utilize the PHH3 slide to screen the slide for MFs, thereby reducing the likelihood of omissions. Concurrently, the morphology of the MFs can be examined in the respective H&E-stained slide^38^. This combined procedure aims at a reduction in the overall label error, since the PHH3 stain, if applied correctly, reveals the biological truth. However, one caveat is that PHH3 is more sensitive to early mitotic phases and less sensitive to later mitotic phases of the cell cycle^20^. While late telophase MFs are well identifiable in H&E because of their characteristic morphology, PHH3 labels early prophase MFs which lack mitotic figure morphology and are hence not identifiable in H&E-stained slides alone^38,39^. As a result, the MFs highlighted in slides stained against PHH3 differ from those recognizable in H&E, contributing to an elevated MC, which was observed in studies involving various tumors^18,40,41^.

The use of PHH3 for mitosis recognition has become more widespread in research in recent years, and it has also been proposed for use in grading schemes^42,43^. While the impact on the inter-rater agreement of MF annotation in slides stained against PHH3 has been the subject of various studies^18,20,21,23,37^, the majority of these studies investigated the MC determined in H&E separately from the MC determined in PHH3-stained slides^18,20,21,23^. This differs from PHH3-assisted approaches^14,28,30^, where the use of co-registered double stains is meant to make the information of both stains available to the pathologists simultaneously.

Our hypothesis is that while the PHH3-assisted labeling methodology may result in a high level of agreement among pathologists, it may also introduce a hindsight bias ^44^, i.e., that the reviewers judge potential MF objects differently in H&E after knowing the biological truth through the PHH3 image, even if the respective cell is lacking MF morphology. This could lead to the inclusion of MFs in the dataset that would not have been annotated in the absence of PHH3, even if the annotation was carried out with an utmost level of diligence and using a consensus of multiple experts for each object. If these cells lack MF morphology and are therefore not identifiable in the H&E, an information mismatch occurs as soon as the information of the PHH3 is missing, which leads to an interpretation shift for MF labels. From the perspective of an object detector operating in the H&E, the annotation of those cells can be considered false positives, i.e., an asymmetric label noise. This may consequently impede the performance of the model during training or result in an underestimation of the model’s performance, in particular the recall, if it is evaluated on such a dataset.

We took several steps to investigate our hypothesis. To assess the influence of PHH3-assisted labeling on the annotations of human experts, we conducted an extensive multi-expert experiment. Thirteen pathologists annotated MFs in two phases on twenty region of interests (ROIs) of four tumor types with and without PHH3 assistance. The resulting data set was used to investigate the impact of PHH3 assistance on the pathologists’ agreement. In addition, the MFs newly found by PHH3 assistance were re-evaluated in a post-hoc experiment to find out what kind of cells were newly annotated by PHH3 assistance. In order to investigate the influence of PHH3-assisted labeling on the performance of deep learning models, the data set resulting from the study was used to evaluate different models. The primary contribution of this work is to challenge the assumption that PHH3-aided labeling procedures inherently provide optimal labels for H&E-stained sections without additional steps. Our findings permit the formulation of an annotation scheme and recommendations for the optimal utilization of PHH3 in the annotation of MFs in H&E.

## Results

### Human rater study

A study was conducted with 13 pathology experts to investigate the impact of co-registered PHH3 slides on the annotation of MFs by human experts. The set of images used in the study consisted of 20 ROIs of four different tumor types of which ten were of canine and ten were of human origin. The study had two phases: in the first phase (P1), the experts were asked to annotate MFs only in H&E-stained slides. In the second phase (P2), conducted after a wash-out period, they were asked to repeat the task with PHH3 assistance, i.e., with H&E and PHH3-stained slides available simultaneously. In P2 the experts had three different classes available for annotation, depending on whether an MF was identifiable only in PHH3, only in H&E, or in both stains. By considering only the last two annotation classes, we only focus on those MFs which the experts classified as at least being identifiable in the H&E. Further details regarding the study design and the study dataset can be found in the methods section.

**Fig. 2:**
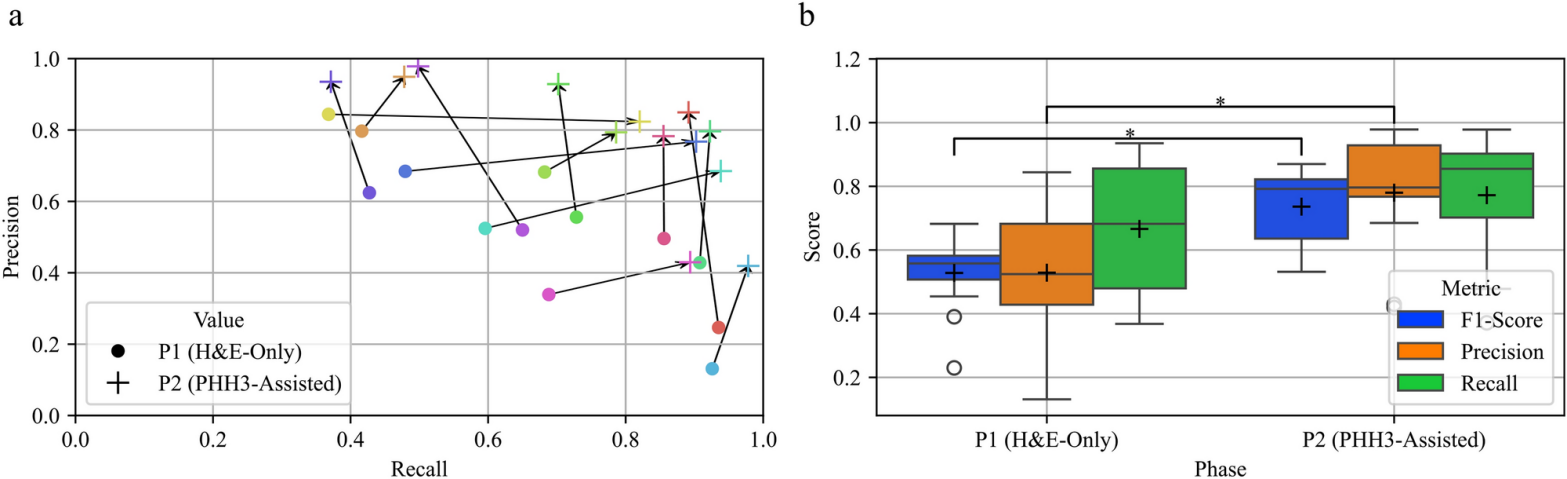
Panel (**a**) displays the precision and recall of each rater plotted against the consensus of the remaining raters of phase one and three. The results of the first (P1) and second (P2) phases of the study are marked by a dot and a cross, respectively. Each rater is represented by a different color. The F1 value, precision, and recall of each rater against the consensus of the remaining raters is given in panel (**b**). Means are indicated by the black crosses. The box represents the interquartile range (IQR) with the median as a line inside it, while the whiskers extend to the smallest and largest observations within $1.5 \cdot$ IQR from the quartiles. A significant difference between the values found in P1 and P2 is indicated by an asterisk.

The agreement of the pathologists was assessed both for the MC and for the individual MF instances. To assess the inter-rater agreement of the MC, we employed the intraclass correlation coefficient (ICC). We computed the ICC for a case in which each target was rated by a fixed set of *k* raters. We then reported the average over *k* ratings, which is equivalent to the Spearman-Brown adjusted reliability. To measure agreement at the object level, we compared each rater’s annotation to the consensus of all other raters’ annotations for each phase of the experiment, resulting in an independent ground truth definition for each of the two study phases. We recognized MFs in our ground truth if they were recognized by at least half of the remaining experts (i.e., six). The agreement between the individual raters’ annotations and the described consensus was then measured via Dice Similarity Coefficient/F1-score, as proposed for this task by Veta et al.^13^. Additionally, we calculated precision and recall (see Fig. 2).

The usage of PHH3 assistance resulted in a substantial improvement in instance-level agreement between raters (see Fig. 2). In Fig. 2a the precision and recall values for the H&E-based annotation (P1) and the PHH3-assisted annotation (P2) for each rater are plotted against each other. Each rater demonstrated an increase in either recall, precision, or both when comparing the results of phase P1 to P2. If there was a slight decrease in either precision or recall in phase P2, it was always accompanied by a substantial increase in the other metric. The aggregated results over all raters are given in Fig. 2b. Each metric increased by a large margin through the use of PHH3 assistance. In particular, we found that the average F1-score increased from $0.53 \pm 0.11$ to $0.74 \pm 0.11$, the average precision from $0.53 \pm 0.20$ to $0.78 \pm 0.17$, and the average recall from $0.67 \pm 0.19$ to $0.77 \pm 0.19$. For the F1-score and the precision, this difference was found to be statistically significant ($t(12) = -5.86, p<0.05$ and $t(12) = -5.18, p<0.05$). Details about the statistical tests are given in the methods section.

The investigation of the agreement of the MC demonstrated that the annotations performed using only the H&E-stained slides yielded an ICC of 0.9. Nevertheless, the utilization of PHH3-assisted labeling in P2 led to an ICC of 0.99, thereby achieving a nearly perfect level of agreement.

The average number of MFs found per expert per image increased from $20.48 \pm 19.25$ in phase P1 to $29.00 \pm 14.25$ in phase P2. The total number of MFs upon which at least six raters agreed increased from 252 in P1 to 549 in P2. In Phase P2, 308 MFs were newly annotated while 11 MFs annotated in phase P1 were not annotated as MFs in phase P2 by at least six experts.

To investigate the morphology of the MFs that were newly discovered by PHH3-assisted labeling, these MFs were re-assessed in a post-hoc experiment involving three board-certified pathologists. Each newly discovered MF was independently reviewed by the pathologists in solely the H&E image in a blinded experiment. The objective of the experiment was to determine whether a given cell displayed distinct MF morphology in the H&E or not. In case the experts reached a differing conclusion, the class was determined by majority vote. More details of the experiment design are provided in the methods section. Of the 308 newly found MFs in condition P2, 172 were classified as not being recognizable based on the H&E-stained image alone by at least two of the three experts. The examination of the agreement of the pathologists in this decision showed a low agreement with a Fleiss’ kappa value of 0.20, which highlights the difficulty of this task. Further analysis of the 308 newly found cells by an experienced pathologist showed that the majority ($48\,\%$) represented prophase or prometaphase, $37\,\%$ Metaphase, and $15\,\%$ anaphase or telophase.

### Deep learning experiment

As shown above, the inter-rater agreement increased significantly if the experts were aided by the secondary stain of PHH3, and consequentially, we can expect a reduction in the dataset label noise. It is therefore interesting to observe which effect this increased consistency in the training and evaluation dataset has on deep learning pipelines.

We evaluated two different state-of-the-art object detection architectures on datasets derived from the human rater experiment (termed *study dataset* in the following) and another independent dataset. We used the Fully Convolutional One-Stage Object 165 Detector (FCOS) by Tian et al.^45^ as a single-stain detector based solely on H&E staining. This architecture was then extended with a second feature extractor, as shown in Fig. 5, to function as a dual-stain detector (termed Dual-Input FCOS (DI-FCOS) in the following). The training of the described detectors required a dataset which had two different ground truth definitions available, one based on H&E and the second based on PHH3-assisted labeling. For this purpose, a sub-set of the final test set of the 2022 edition of the MIDOG challenge was used, to which we will refer in the following as the *MIDOG* dataset. The dataset has been annotated using a consensus of three raters both using only the H&E images and, additionally, using PHH3 assistance^28^.

To increase the statistical informativeness of the results we trained each object detector in a five-fold Monte Carlo cross-validation scheme. For this, the MIDOG dataset was randomly split five times into $70 \%$ training, and $15 \%$ each for validation and test cases. To ensure comparability of the results, all models were trained and tested on the same five splits. Besides on the test split of the MIDOG dataset, the detectors were also evaluated on the *study dataset* derived from P1 and P2 of the human rater experiment. We used the average precision (AP) to measure the performance of the object detection algorithms.

For DL models receiving H&E images as input (i.e., single-stain models), we evaluated the use of PHH3-assisted annotations in both training and evaluation, and contrasted these results against training and testing with annotations generated on solely H&E images, all by multi-expert consensus.

Firstly, we found that the performance of the single-stain detectors decreased when evaluating on the PHH3-assisted annotations, regardless if we trained on the H&E-only annotations or the PHH3-assisted annotations (see Table 1). We found this result consistently for both independently annotated datasets (MIDOG dataset and study dataset). The single-stain detectors hence do not benefit from the improved consistency in the evaluation dataset.

Secondly, we found that training on the PHH3-assisted label sets does not improve the performance of the single-stain detectors, regardless on which dataset these were evaluated. Single-stain detectors are thus not able to leverage the improved consistency in the training dataset, independent of which evaluation set was used.

Furthermore, the overall best performance for the single-stain detectors was found when training and evaluation was done on labels generated as consensus of experts solely on H&E stain.

In summary, these results indicate that H&E-based detectors do not benefit from PHH3-assisted labels, whether they are used in the training or evaluation sets.

The situation changes when we allow the detectors to also utilize the additional PHH3 image (DI-FCOS condition in Table 1). By providing the model with the same information available to human annotators, the network significantly improves and achieves top scores on both independently collected datasets. This points directly to an information mismatch between the condition where only one stain is available and when both stains are available, leading to an interpretation shift as to which objects can be identified as MFs. We should note that we explicitly asked annotators to not annotate any objects that did not have clear morphological features. Hence, this indicates that PHH3-assisted labeling imposes a hindsight bias on the annotating experts, i.e., the presence of PHH3 markers inadvertently influences their judgment and causes over-recognition of cellular objects that have no clear MF morphology in H&E^44^. This leads to the inclusion of MFs in the dataset that would not have been annotated without PHH3 assistance, as they lack clear MF morphology in the H&E staining.

**Table 1:** Results of the single stain (FCOS) and dual stain (DI-FCOS) object detection models on the different label sets of the test split of the MIDOG dataset and the 13 expert study dataset, evaluated in five-fold cross-validation.

Test dataset	Model	Stains	Parameters	Trained using H&E-only labels		Trained using PHH3-assisted labels	
				Evaluation labels		Evaluation labels	
				H&E-only	PHH3-assisted	H&E-only	PHH3-assisted
MIDOG dataset	FCOS (ResNet18)	H&E	19.0 M	$0.70\pm 0.03$	$0.64\pm 0.02$	$0.71\pm 0.03$	$0.68\pm 0.04$
	FCOS (ResNet101)	H&E	51.0 M	$\mathbf {0.74}\pm 0.04$	$0.68\pm 0.05$	$0.71\pm 0.04$	$0.69\pm 0.06$
	DI-FCOS (ResNet18)	PHH3,H&E	39.9 M	$\mathbf {0.74}\pm 0.04$	$0.73\pm 0.05$	$0.72\pm 0.04$	$\mathbf {0.79}\pm 0.05$
Study dataset	FCOS (ResNet18)	H&E	19.0 M	$0.64\pm 0.02$	$0.58\pm 0.03$	$0.61\pm 0.04$	$0.61\pm 0.02$
	FCOS (ResNet101)	H&E	51.0 M	$0.66\pm 0.03$	$0.60\pm 0.02$	$0.62\pm 0.03$	$0.60\pm 0.04$
	DI-FCOS (ResNet18)	PHH3,H&E	39.9 M	$0.66\pm 0.04$	$\mathbf {0.72}\pm 0.06$	$0.61\pm 0.05$	$\mathbf {0.81}\pm 0.05$

Given are the mean and standard deviation of the average precision (AP) as a result of cross validation. Best results for each dataset and each training setting are highlighted in bold.

We found additional evidence of an interpretation shift when comparing the dual-input DI-FCOS models (which can utilize both stains) between models trained on the PHH3-assisted and the H&E-only labels. If and only if the dual-stain models were trained and evaluated on the PHH3-assisted labels, we found a superior performance (see bold-marked results in right column of Table 1). Training on the H&E-annotated labels strips this detector from a part of its competitive advantage (average APs dropping from 0.81 to 0.72 (study) and 0.79 to 0.73 (MIDOG)). To investigate a possible effect of the dual-stain DI-FCOS model just having a greater capacity and thus being able to perform better, we also investigated the single-stain approach using a much larger backbone (ResNet101 vs. ResNet18), which more than doubled the number of parameters of the model and exceeded the number of parameters of the dual-stain model by a considerable amount. The similar performance of the DI-FCOS model and the FCOS model with the larger backbone on the H&E-only label sets, along with the superior performance of the DI-FCOS model on the PHH3-assisted label sets, indicates that the superior performance of the DI-FCOS model was not merely due to its larger model size.

**Fig. 3:**
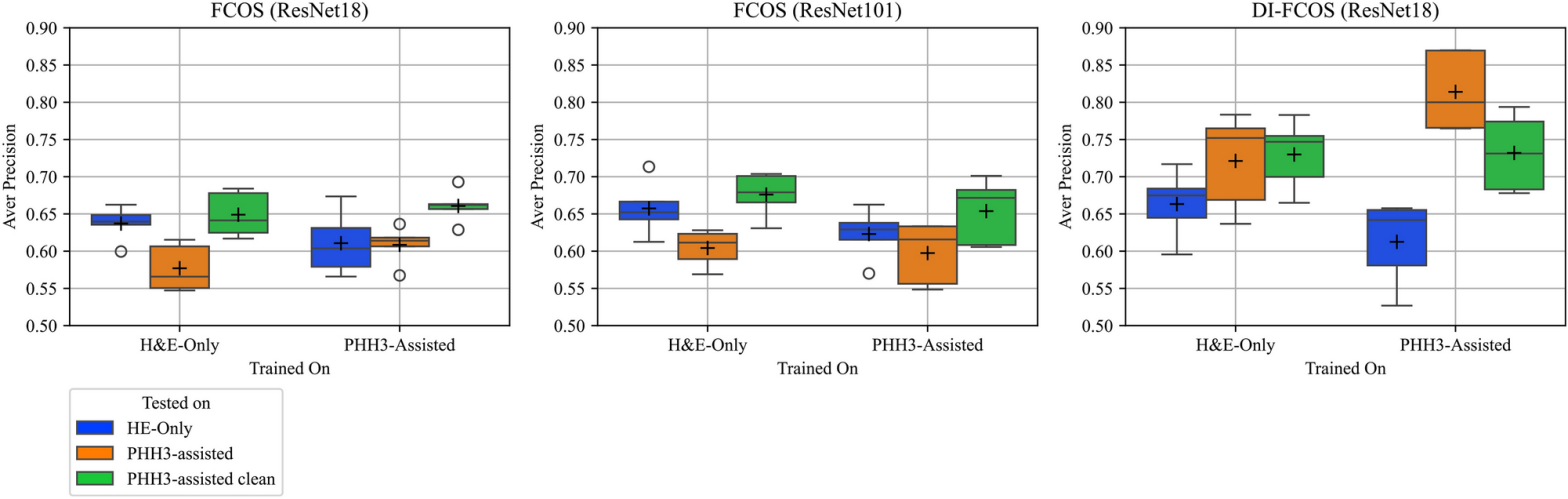
Average precision of the single-stain FCOS and dual-stain DI-FCOS models on the test sets, evaluated in a five-fold cross-validation on the label-sets of the study dataset. Boxes visualize the first quartile and third quartile, lines indicate median and pluses indicate mean values of the runs.

To further substantiate our analysis regarding the hypothesis that the PHH3 assistance caused the inclusion of MFs into the dataset that lack MF morphology and are hence not identifiable by a single-stain detector solely based on H&E, we ran another experimental evaluation. In it, we used the three expert consensus from the post-hoc experiment to create a further, cleaned label set for the study dataset derived from P2, excluding the 172 MF cells which the three experts classified as non-recognizable in the post-hoc analysis. The evaluation of the single-input detectors on the cleaned version of the PHH3-assisted label sets revealed the best results for these detectors (see Fig. 3). Compared to the results on the PHH3-assisted (evaluation) label sets, the performance increased by a large margin. This aligns with our hypothesis that the single-stain detectors were unable to detect MFs in the PHH3-assisted dataset due to a difference of available information between the H&E- and PHH3-stained images, leading to an interpretation shift for MF labels. The exclusion of the cells lacking MF morphology presumably reduced the effect of the interpretation shift and led to better performance of the single-input detectors. We did not observe the same performance gains for the dual-input models. Specifically, the performance of the DI-FCOS model trained on a PHH3-assisted label set deteriorated when evaluated on the cleaned version of the PHH3-assisted label set. This supports our assumption that the dual-input detectors were not subject to the information mismatch between labels and image information like the single-input detectors. Otherwise, the exclusion of the unclear MFs should have also positively affected the dual-input detectors.

**Fig. 4:**
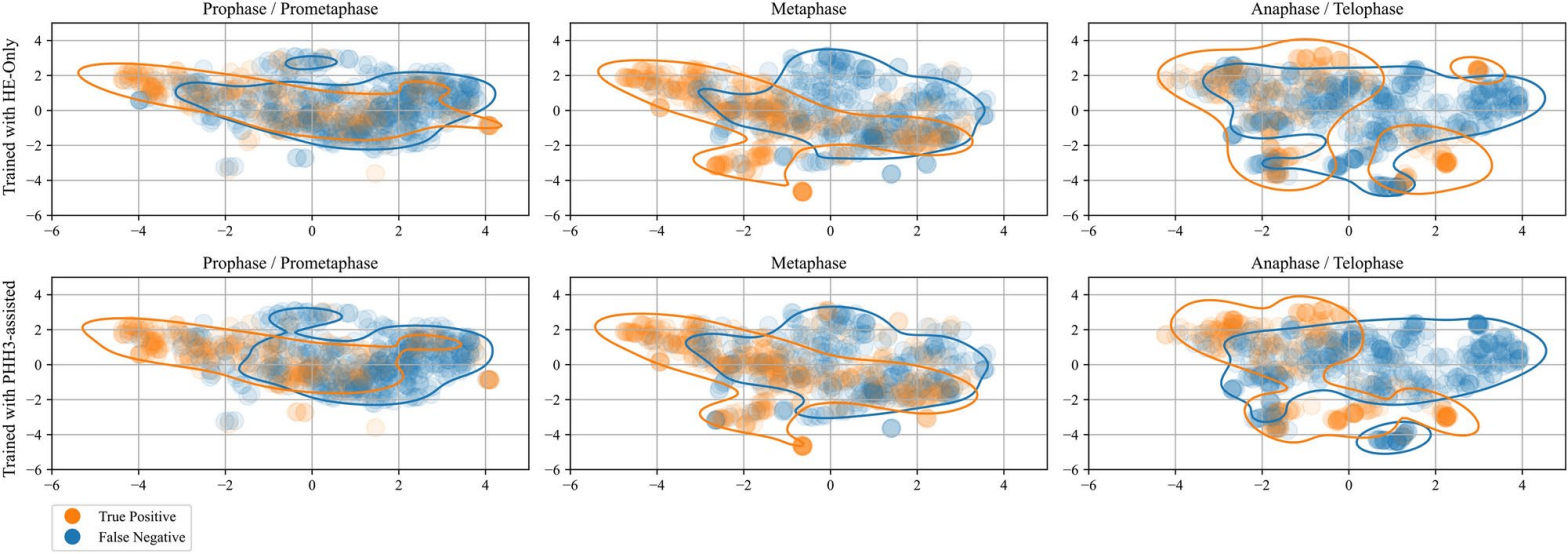
A UMAP representation of the latent space of a MF classifier at varying stages of mitosis, showcasing a shift of the decision boundaries for the prophase/prometaphase. The projections are colored according to whether a respective cell was identified by the majority of the models included in the respective cross-validation and thus constituted a true positive (orange) or not (blue). The first row depicts the decisions of the ResNet18-based single stain detector, which was trained with H&E-only labels. In contrast, the second row illustrates the decisions based on the same architecture but trained with PHH3-assisted labels. To facilitate a more comprehensive visualization of the distributions, the orange and blue lines demarcate the region encompassing the three quartiles of the respective true positive and false negative decisions.

To further examine the impact of training with H&E-only and PHH3-assisted labels on the decision of single input detectors, we employed a Uniform Manifold Approximation and Projection (UMAP)-based^46^ visualization approach on the latent space embeddings of the 308 MFs from the study dataset for which we had information available about the mitotic phase (see Fig. 4). To achieve a sensible discrimination of the MFs into the latent space, a single mapping network was trained on the MIDOG dataset. We then projected the latent space of this network into a two-dimensional space by means of the UMAP projection.

The UMAP projections were then color-coded depending on whether the majority of the models of the respective cross validation found a respective cell which thus constituted a true positive or whether a cell was not found and hence constituted a false negative. We assume that this way we are able to visualize the effect of the labeling method on the decision boundaries of the respective models trained using the labels acquired with this labeling method. Further details on the training of the mapping network and the visualization process are provided in the Methods section.

In our projection the discrimination between recognized and non-recognized MFs is predominantly along the horizontal axis. A clustering of true positives and false negatives is visible for each plot. We also visualized the 75th percentile of the respective distributions in Fig. 4. Comparing the distributions of false negative predictions of prophase and prometaphase MFs for the models trained with H&E-only labels and PHH3-assisted labels, a shift of the decision boundary along the horizontal axis is visible comparing the PHH3-assisted vs. the H&E-based models. There is no such shift present for the other mitotic phases. This is an indication that training with PHH3-assisted labels shifted the decision boundary towards MFs which were harder to identify only for prophase/prometaphase MFs.

## Discussion

Reliable and robust MF detectors can be of great help in computer-aided tumor grading. High label quality is crucial for the training and evaluation of such DL-based detectors, but is particularly difficult to achieve given the low inter-rater agreement and reproducibility^13^. Previous studies have attempted to resolve this ground truth problem by utilizing co-registered slides, re-stained against PHH3, an IHC stain that specifically highlights MFs. Those PHH3-stained slides were given to the pathologists either as a sole stain or alongside the H&E-stained slides to avoid omissions of MFs in the H&E-stained slides and to enhance the inter-rater agreement^25–31^.

A fundamental, yet so far untested, assumption about PHH3-assisted labeling was that, given the reported higher inter-rater agreement on an object level, the resulting definition of a ground truth (with regard to the H&E-stained images) is of much higher quality than annotations on H&E alone. An improved labeling consistency should reduce the label noise, benefiting the evaluation metrics if used for acquisition of the test set, and also benefiting the prediction quality of the models trained with such data. Given this, we would thus expect to find improved metrics if trained or evaluated on the PHH3-assisted labels.

However, we found the opposite to be true in our study. On two independently collected datasets, we found the AP metric to decrease if we train or evaluate single-stain detectors on PHH3-assisted labels. The use of PHH3-assisted annotation workflows imposes an information mismatch, leading to an interpretation shift of what constitutes a mitotic figure. This ultimately leads to the inclusion of MFs into the H&E dataset which would not have been annotated without the PHH3 assistance available, as these MFs are hard to recognize in H&E-stained images alone. While some objects might be borderline identifiable (leading to a semantic label shift inconsistent with H&E-based annotations), other objects might not be distinguishable at all (introducing label noise if only the H&E image is considered).

The introduction of such unrecognizable MFs is evidenced by the results of the post-hoc analysis of MFs that were newly annotated in the PHH3-assisted annotation setting. The majority of those newly annotated cells were classified as being not recognizable as MFs with H&E due to the absence of characteristic morphological features. Excluding these cells from the PHH3-assisted label set resulted in an improvement in the evaluation performance of the H&E-based single input detectors (see Fig. 3), which demonstrated their negative influence on the the evaluation results.

Furthermore, our investigations indicated that training single-input detectors with PHH3-assisted labels shifted the decision boundary for prophase and prometaphase MFs, skewing it towards less distinct MFs (see Fig.4). This shift was likely due to the inclusion of early prophase MFs through PHH3-assisted annotations, which were not easily identifiable in H&E staining. Given that prophase is one of the longest phases of the mitotic cycle^38^, it is expected that the use of PHH3-assisted annotations increases the representation of this phase within the dataset, potentially driving the observed shift in the model’s decision threshold.

The discrepancy in information between the H&E- and PHH3-stained images is further indicated by the superior performance of the dual-input detectors (see Table 1). In contrast to single-input detectors, the dual-input detectors have access to the same information as the annotating experts. Consequently, from the perspective of the dual-input detector, there is no information shift. The information shift occurs only when the information from the PHH3-stained slide is absent, as it is from the perspective of the single input detectors. This ultimately resulted in the lower performance of the single-input detectors on the PHH3-assisted label sets.

The substantial difference between dual-stain detectors trained on the PHH3-assisted labels and those trained on H&E-based labels emphasizes this interpretation: We can conclude that this might be indicative of the dual-stain detector learning to classify early prophase and other morphologically indistinguishable cells as MFs only if it was trained on the PHH3-assisted label set, which includes these objects. If trained on the H&E-based annotations, while having the PHH3 staining information available, the detector will learn to classify those objects as non-MFs. This interpretation is supported by our results, where the average AP considerably drops when trained on H&E-based labels alone.

We found a relatively low level of agreement among our experts in the post hoc experiment as to whether or not a given cell exhibited morphological characteristics of an MF. Since the experts involved in this experiment were all board-certified pathologists with extensive experience in identifying MFs, we do not attribute the low agreement to a lack of expertise. Rather, we attribute the low agreement to the difficulty of the task. The cells given to the experts in the post-hoc experiment were those identified by the majority of pathologists in the annotation experiment only after PHH3-assistance. Therefore, it can be assumed that these did not generally show conspicuous MF morphology, representing borderline cases. This is corroborated by the subsequent assessment of the newly discovered cells, which revealed that the majority of cells were either in prophase or prometaphase. A particularly low agreement between pathologists is reported for these phases, which is why it has already been recommended not to count them when determining the MC^38,39^.

A limitation of our DL experiments is that a DL model serves only as an indirect measure of information availability. If the information is not present, the DL model cannot utilize it. However, the absence of improvement in a DL pipeline cannot be solely attributed to a lack of additional meaningful information that could be utilized for the decision. To the contrary, the model could just not be using the additional information. The high concordance between all DL results and the statistical evaluations conducted by human participants reassures us that this limitation was not significant within our study. Furthermore, the dataset only contained 84 ROIs which is relatively small compared to the size of other datasets for MF detection like the training set of the MIDOG 2022 challenge^28^ or even datasets consisting of completely annotated WSIs^47^. Since the focus of this work was to investigate the information mismatch between H&E- and PHH3-stained slides rather than developing an MF detector with competitive performance, we consider this as a minor limitation. Additionally, at least for the H&E part of the dataset, all compared detectors (FCOS using ResNet18, FCOS using ResNet 101, DI-FCOS) were trained on the same physical slides, so it can be assumed that they were all equally influenced by the available data variance.

Since the IHC label present in the PHH3-stained slides is dependent on the biological process of cell division, the PHH3-assisted ground truth can be considered a more accurate estimate of the actual biological truth. Therefore, an MF detector trained with PHH3-assisted labels in H&E images alone could be similarly perceived as biologically more accurate. However, if the biological information is not available in the H&E slides, as indicated by our work, the detector will perform less accurately. If the cutoff is optimized on the PHH3-assisted annotations, it is likely that the detector will include those doubtful MF candidates, and also other morphologically similar objects, reaching a high false positive rate. Ultimately, the goal of MF detection in digital pathology is commonly to determine the MC for use in grading schemes. The current schemes are also based on solely H&E stains^3–12^. Hence, while the PHH3-assisted label set might represent the biological truth of cell division more accurately, it reflects different boundary conditions than those present in routine oncologic histopathology.

Our findings demonstrate that PHH3-assisted annotation significantly enhances the object-level agreement of MFs among experts. While this was the first time the impact of PHH3-assisted annotation was investigated, these results align with previous studies comparing the inter-rater agreement of MF counting using H&E with the inter-rater agreement of MF counting using PHH3^11,18,20,23,37^. One possible reason for this might be that the experts overlooked fewer MFs using the PHH3-assisted pipeline. This is supported by the higher average number of MFs identified per expert per slide and the recall rate for individual objects, which increased by the use of the PHH3s-assisted annotation procedure. The significant increase in precision also shows that the experts agreed more on the objects they annotated, as the individual expert made fewer false positive annotations in relation to the ground truth formed by the rest of the experts. This indicates that the IHC label in PHH3 provided a strong decision criterion to which the experts adhered to.

Although Fig. 2a may suggest that some experts improved more in precision while others showed greater gains in recall, this cannot be attributed to differences in how the experts utilized the PHH3 assistance, as all experts underwent the same training prior to the second stage of the human rater experiment. Therefore, it is reasonable to assume that the observed discrepancies are due to variations in the internal cutoff values applied to MF morphology and IHC labeling, as well as the relative weighting of these factors in the raters’ decision-making processes.

The tissue preparation utilized in this study is considerably more complex and costly in comparison to the conventional processing using only H&E staining. Additionally, re-staining of H&E sections is not a routine procedure, which also carries the risk of damaging the tissue, making it challenging to register the resulting slides and requiring the entire preparation procedure to be repeated. Furthermore, both sections must be scanned and the data stored, which further increases the demands on the computational resources. It is therefore unlikely that this method, or the double staining proposed by Ibrahim et al.^37^, will replace the routine counting of MFs in H&E-stained sections. However, it can be of high value in the annotation of MF datasets used to train and evaluate automatic detection pipelines, if the PHH3-positive and morphologically doubtful annotations are excluded post-hoc, as this study shows (see Fig. 3).

For this reason, we propose an adapted annotation procedure based on our results. Given the high recall of the PHH3-assisted annotation procedure, the double-stained sections could be utilized in an annotation system to identify potential MF candidates in an initial step, potentially also only by a single expert. Consequently, the PHH3-assisted annotation procedure could potentially replace complex AI-assisted annotation methods^16,28^. As the visual contrast between MFs and the surrounding tissue is considerably higher in slides stained against PHH3 than in those stained with H&E, screening for MFs can be considered less tiring and faster. Since the cells that carry a positive signal in the IHC biologically represent mitosis, the possibility of accidentally labeling non-MFs is also reduced. While cells that show both a clear IHC signal and very clear MF morphology in the H&E can be directly included in the data set, all other identified cells would be classified as MF candidates. Those MF candidates should be forwarded to a panel of experts for independent evaluation, as was done in the post-hoc experiment in this study. The experts will determine whether the respective MF candidate can be recognized as an MF in the H&E-stained slide. In this assessment, the experts should utilize the same morphological criteria to inform their decision as for example those described by Donovan et al.^38^. To mitigate potential cognitive biases introduced by the availability of the PHH3 stain, the experts should perform this task solely with the H&E stains available. Additionally, some MF lookalikes should be included in the cells in this assessment to prevent indirect influences due to hindsight bias. If the expert panel was only presented with MF candidates that were positive in the IHC, this knowledge could bias them towards a lower internal threshold regarding the morphological features, which in turn could again lead to the inclusion of MFs in the dataset that do not have a distinct MF morphology. The cells which a majority of experts recognize as MFs can then be included in the data set.

Our study represents conclusive evidence that PHH3-restained slides, co-registered with H&E slides can be of great use in the efficient and accurate annotation of MFs. However, our results also imply that the direct application of these labels to H&E leads to an interpretation shift, as a considerable number of objects that are positive in PHH3 are not distinguishable in H&E, which can be interpreted as label noise in the context of the H&E image. We propose to remedy this problem with a secondary evaluation and consensus by a panel of experts using image patches. Since this is a relatively expeditious task, we are optimistic that these steps could represent the new gold standard for mitotic figure annotation.

## Methods

### Dataset description

Two different datasets were used in this study, one for the training and evaluation of the object detectors and one generated by the human rater experiment, which serves as a hold-out dataset for the evaluation of the object detectors. Both datasets utilize corresponding ROI pairs of tumor tissue stained with two stains as depicted in Fig. 1a: The source slides were initially stained with H&E, digitized, and subsequently de-stained and re-stained with PHH3 before being digitized again. The resulting WSIs were then registered using a registration algorithm for WSIs^48^. From the co-registered pairs of re-stained original WSIs, the ROIs were selected by two pathologists (C.A.B. and S.J.) following the criteria outlined in the grading schemes^4,11,49^, and excluding areas that might be of insufficient tissue, scan or stain quality in either stain, or areas where loss of tissue occurred in the second stain.

#### Image dataset for human rater study

The dataset used in the annotation study consists of 20 ROIs representing four different tumor types, two of which were of human origin and two of veterinary origin. Tumors of different tumor types and species were included in order to draw broader conclusions from the results of the study. The MC is included in the respective grading scheme of each tumor type included in this study^4,11,49^. Five samples each of human astrocytoma and meningioma were collected from the diagnostic archive of the *Institute of Neuropathology, University Hospital Erlangen, Germany*, after prior ethics approval by the institutional review board (Ethik-Kommission der Friedrich-Alexander-Universität Erlangen-Nürnberg, AZ 92 14B, AZ 193 18B). Slides containing animal tissue were retrieved retrospectively from the diagnostic archive, which requires no ethics approval according to local laws and regulations. Informed consent was obtained from all subjects and all methods were carried out in accordance with relevant guidelines and regulations. The slides were digitized using an Hamamatsu NanoZoomer S60 at $40\times$ magnification. From the diagnostic archive of the *Institute of Veterinary Pathology, University of Veterinary Medicine, Vienna, Austria,* five samples each of canine cutaneous mast cell tumor (CCMCT) and of canine mammary carcinoma (CMC) were collected. For these slides, no ethics approval is needed, according to local laws and regulations. All specimens were originally acquired for diagnostic reasons. These veterinary slides were digitized with a 3DHistech Pannoramic Scan II at $40\times$ magnification. Each ROI was selected to cover $2.37\,\hbox {mm}^2$ of tissue, which is equivalent to approximately ten high power fields^2^. In the remainder of this paper we refer to the dataset generated by annotating these images as *study dataset*.

#### MIDOG dataset

To examine the impact of PHH3-assisted MF annotation on the performance of MF detectors, we trained detectors with annotations acquired only using H&E-stained slides and with annotations that were generated through PHH3-assisted annotation. For this purpose, a sub-set of the final test set of the 2022 edition of the MIDOG challenge^28^ was used. The dataset contained ten samples of CCMCT, nine samples of CMC, ten samples of canine hemangiosarcoma, nine samples of feline lymphoma, ten samples of feline soft tissue sarcoma, three samples of human astrocytoma, ten samples of human bladder cancer, nine samples of human colon carcinoma, ten samples of human melanoma, and four samples of human meningioma. We refer to this as the *MIDOG dataset*. Two different ground truth definitions were available for this dataset. The first definition relied solely on H&E-stained slides, while the second was created with PHH3-assisted labeling. The H&E-only annotations were created as described in previous works^16^ by three pathologist with at least five years of experience in MF identification assisted by a DL model with high recall that screened the slides for MF candidates. To be accepted as an MF, at least two pathologists had to agree upon a candidate. The PHH3-assisted annotations were created by a single expert using an open source web-based annotation server^50^ where the two corresponding stains could be superimposed on each other with variable transparency as depicted in Fig. 1b. This way, the expert was able to assess the IHC label present in the PHH3-stained slide and the morphological features visible in the H&E slide at once. If the registration was not perfect, e.g., due to tissue deformation, the position of the respective cell in the H&E-stained slide was annotated. Cells that had a positive IHC label but that were lacking MF morphology in the H&E stain were not annotated, as these cells are not identifiable as MFs in the H&E-stained slides^28^. This also covers the case of out-of-focus MFs in the H&E image, which would also lead to an information mismatch if MFs identified solely in PHH3 were used as annotations for the H&E image.

### Human expert mitotic figure annotation study

To investigate the impact of co-registered PHH3 slides on inter-rater agreement, a study was conducted with 13 pathology experts. The study consisted of two phases, with a four week washout period to prevent participants from recalling the cases. To further prevent bias, the slides were presented in randomized order and under different names in each phase. The whole study was conducted using the online annotation tool EXACT^50^ (see supplementary Figure S 4). During the initial phase, participants only had access to H&E-stained ROIs. In the subsequent phase, participants could overlay co-registered PHH3-stained ROIs on the H&E-stained images with adjustable transparency, allowing for simultaneous examination of both stains. Additionally, participants were given three different labels to choose from when annotating MFs in the second phase, depending on whether an MF was identifiable in both H&E and PHH3, only in H&E because of a lacking IHC signal in the PHH3, or only in PHH3 because of unclear MF morphology in the H&E. In all experiments in this paper, only annotations of the first two classes are considered. , i.e., only cells showing clear MF morphology in H&E .This means that only cells where the annotators perceived a clear MF morphology in the H&E were included into our definition of ground truth. In case of imperfect registration, participants were instructed to annotate the position of the respective cell in the H&E-stained image. Before the second phase a pathologist highly experienced in PHH3-assisted labeling (C.A.B.) conducted an online training with the participants.

To build the consensus set of agreed-upon mitotic figure objects, we used a distance-based clustering approach. An annotation was added to a cluster if it was no more than 7.5 micrometers away from the center of the cluster, approximately corresponding to the diameter of a nucleus^51^. This was done since the object coordinates determined by individual raters might vary significantly, and we wanted to avoid attribution of MF objects to multiple consensus annotations.

The cluster centroid was accepted to the consensus set as MF if it contained MF annotations from at least six experts. This threshold was chosen because the instance-based metrics were computed in a one-versus-all fashion. Specifically, if the instance-level agreement for one expert was to be computed, the remaining twelve experts were used to generate a consensus set. Thus, a threshold of six effectively indicates that at least 50% of the expert group agreed on the identification of an MF.

Setting the consensus threshold at six experts was considered appropriate due to the nature of the task. In object detection, especially for challenging objects such as MFs, raters can often miss detections. Previous studies have shown that even expert raters tend to overlook difficult-to-identify MFs^13,16^. Therefore, requiring a higher number of experts to agree might lead to the exclusion of legitimate MFs, thus reducing the overall detection performance. Although the consensus threshold is a hyperparameter that can influence the results, our observations indicate that similar trends persisted across various thresholds (see supplementary Figures S1 and S2).

To assess the statistical significance of the discrepancy between the instance-based metrics observed across the two study phases, we employed a paired t-test. The normality of the differences was evaluated using a Shapiro-Wilk test. Given that we conducted a test for each metric, we also applied a Bonferroni correction to minimize the occurrence of Type I errors by dividing the significance threshold by the number of tests. All statistical evaluations were carried out using the scipy python package, version 1.10.1.

To assess the morphology of the MFs that were annotated by the majority of the experts only with PHH3 assistance, the MFs that were newly found in the second phase of the study were re-assessed in a post-hoc experiment by three board-certified pathologists with high expertise in MF identification (C.A.B., T.A.D., and R.K.). The experts determined whether a respective cell (MF-candidate) was recognizable as an MF based on its morphological features visible in the H&E. To reduce a bias introduced by a possible signal in the PHH3, the experts re-evaluated the cells only based on the H&E-stained slides. Additionally, a certain number of mitotic figure lookalikes were added to the cells that the pathologists examined. This was done to prevent the experts from assuming that a cell in question had a positive IHC label in any case, as it had been annotated in the second phase of the study, which might have introduced a bias on the experts. In total, two-thirds of the cells that were given to the experts were newly annotated cells, while one-third were lookalikes. However, the experts were unaware of this distribution.

Afterwards, the 308 newly found cells were reevaluated by a single expert (T.A.D) to classify them into the different phases of cell division.

**Fig. 5:**
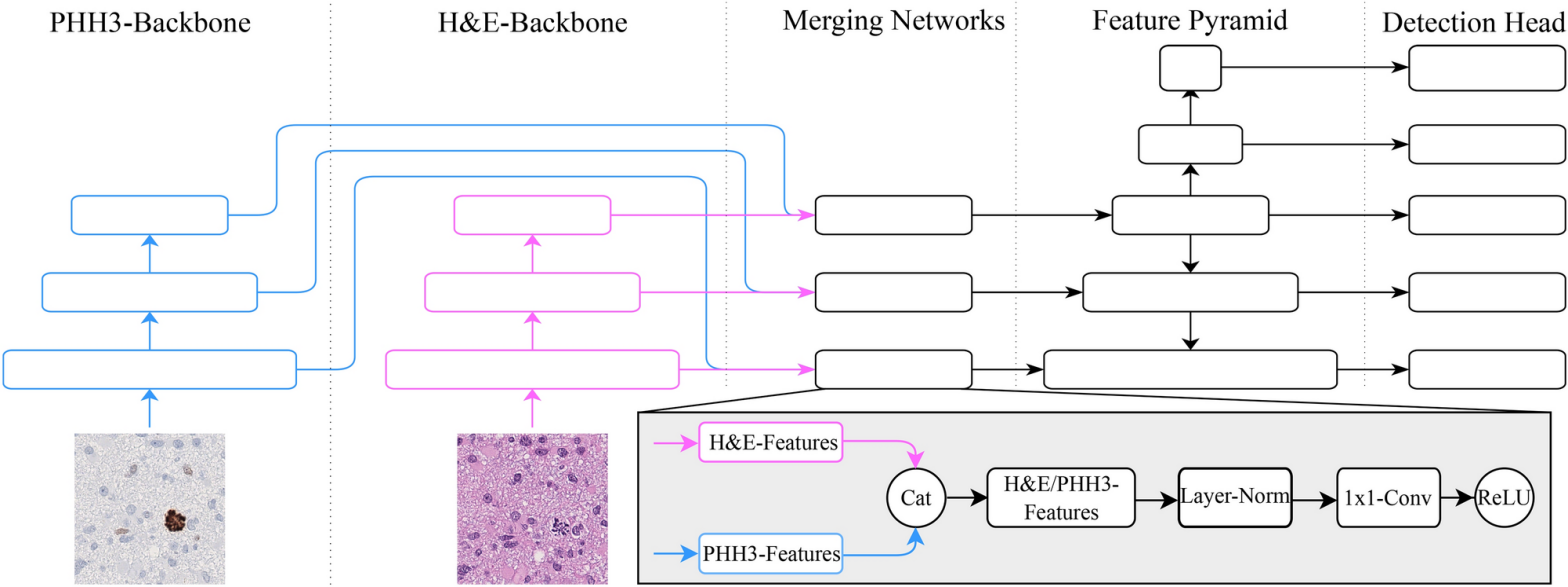
Architectural overview of our dual-stain MF detector. We extended the FCOS architecture by a secondary feature stem for PHH3, fused by mid-fusion using a dedicated fusion network.

### Detection architectures

To assess the impact of PHH3-assisted labeling on the performance of object detectors, we used the Fully Convolutional One-Stage Object Detector (FCOS)^45^, which has already been successfully used to solve other detection tasks in digital pathology^52–54^. Unlike other popular object detection architectures like Faster R-CNN^55^ or RetinaNet^56^, FCOS does not rely on anchor boxes and hence comes with a reduced set of hyperparameters to tune.

To serve as a dual-input object detector, we extended the FCOS detector as depicted in Fig. 5 by adding a second feature extraction branch. This modification enabled the model to be trained on corresponding H&E and PHH3 patches simultaneously, by forwarding the corresponding patches through the respective feature extraction branches of the network. As in the single-input FCOS model, we used the stem of a ResNet18 pre-trained on ImageNet in each of the feature extraction branches. Each feature extraction branch generates a distinct feature embedding for each of the corresponding image patches. To integrate the two feature embeddings into a unified feature embedding that encompasses the information from both input modalities, mid-fusion is employed. This refers to a technique where different input modalities are combined at an intermediate stage within the network^57^. In particular, the features of each ResNet level are fused before they are forwarded to the feature pyramid, using one merging network for each input level of the feature pyramid. Let $\textbf{H}\in \mathbb {R}^{C \times H \times W}$ and $\textbf{P}\in \mathbb {R} ^{C \times H \times W}$ be the feature maps of a level of the H&E and PHH3 backbone, where *C* represents the number of channels of the respective level and *H* and *W* are the sizes of the feature maps which depend on the size of the input image. Then the fused features $\textbf{F}\in \mathbb {R}^{C \times H \times W}$ are computed by $\textbf{F} = \text {{ReLU}}(\text {{Conv}}(\text {{LayerNorm}}(\text {{Cat}}(\textbf{H}, \textbf{P}))))$, where $\text {Cat}$ denotes a concatenation of the feature vectors along the channel dimension and Conv is a $1 \times 1$ convolution which halves the number of input channels to *C* after the concatenation. The rest of the network follows the standard FCOS architecture as described by the authors^45^. To confirm that a difference in model performance between the FCOS and DI-FCOS models is not due to the DI-FCOS models’ higher number of parameters due to its second feature extraction branch, we also compare it to an FCOS detector with a ResNet101 backbone pre-trained on ImageNet. For all experiments, the feature maps from the second, third, and fourth blocks of the respective backbone were used to construct the feature pyramids for both the standard FCOS and the DI-FCOS model. We used a fixed learning rate of $10^{-4}$ and AdamW as the optimizer. All models were trained until convergence, which was observed using the AP metric on the validation set, which we also used for early stopping and for model selection using a patience of 5. All models were trained on patches with a height and width of 512 pixels at 40x magnification, and patches were selected so that at least $50\,\%$ of the training patches contained MFs. A standard image augmentation pipeline was employed during the training of each object detector in this study. This consisted of four augmentation operations including color jitter, Gaussian blur, pixel drop, and random rotation.

While the validation was conducted on patches which were sampled in a manner consistent with that employed for the training patches, the evaluation of the test images was performed differently. During the evaluation of the test images, the images were divided into overlapping patches of 512 by 512 pixels with an overlap of 50 pixels at 40x magnification, and the detector was applied to these patches. The resulting detections were then transferred back into the coordinate system of the original image, to calculate the AP. As the patches overlapped, duplicate detections were removed after transfer to the original coordinate system using non-maximum suppression. To investigate the decision boundaries of the single-stain detectors, we conducted a UMAP-based feature analysis. A ResNet18 model, trained for MF classification, was used as a mapping network to generate meaningful discrimination in feature space. The mapping network was trained on a random split of training and validation sets from the MIDOG dataset. Once the model converged, as observed by the validation loss, the classification layer was removed, and the trained feature stem was used as the mapping network. During the training of the mapping network, the same augmentation pipeline was employed as used during the training of the detection networks. For the decision boundary analysis, we utilized 308 MFs of the post-hoc analysis, for which the corresponding mitotic phase was known, which allowed us to analyze each mitotic phase separately. To densely populate the feature space and better infer the learned decision behavior of the networks, we employed test-time augmentation while forwarding the cells through the mapping network, yielding a total of 2000 projected MF representations. The same augmentations applied during the network’s training were also used here. Subsequently, the resulting $512 \times 1$ dimensional feature vectors were projected into a $2 \times 1$ dimensional space using UMAP. To visualize the models’ decision boundaries, the projected features were color-coded based on whether the respective MF was detected by the majority of models in the cross-validation, indicating a true positive, or not. Since the same projected feature vectors were used for the plots in each column of Fig. 4, the only variation across the plots in each column lies in the color coding. Distribution densities were calculated for the true positive and false negative distributions to highlight differences. The regions containing three quartiles of each distribution was then marked for clearer visualization. Since we expect a different behavior for the different mitotic phases, we have created one such plot per cross-validation for pro- and prometaphase mitoses, metaphase mitoses and ana- and telophase mitoses.

## Supplementary Information

Supplementary Information.

## Data Availability

The data that support the findings of this study are available on reasonable request from the corresponding author M.A. The data are not publicly available due to being part of the confidential test set of the MIDOG 2022 challenge.
