# Supplementary material for: Information mismatch in PHH3-assisted mitosis annotation leads to interpretation shifts in H&E slide analysis
Source: Sci Rep. 2024 Nov 1;14:26273. doi: 10.1038/s41598-024-77244-6 (PMC11530454; doi:10.1038/s41598-024-77244-6)
Supplement: Supplementary file 1 — Supplementary Information. [file 41598_2024_77244_MOESM1_ESM.pdf]

1 Supplementary Materials

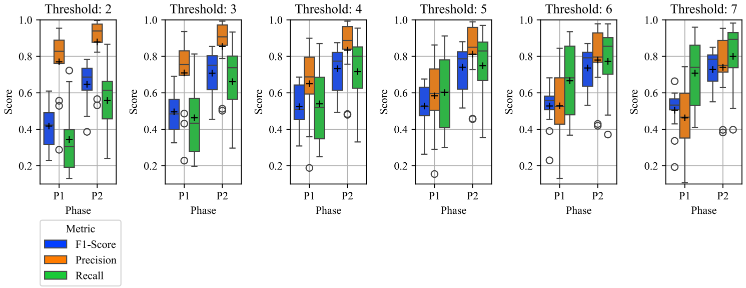

Fig. S1. Comparison of F1-score, precision, and recall for individual raters against the multi-rater consensus. The analysis considers varying thresholds for the number of raters required to agree on a MF in forming a consensus. Higher inter-rater agreement is found for PHH3-assisted annotation (P2) compared to HE-based annotation (P1) for different consensus thresholds.

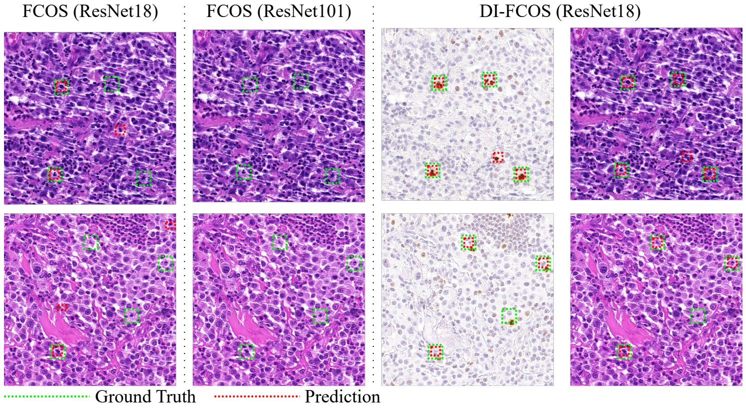

Fig. S2. Qualitative detection examples of the FCOS and DI-FCOS models on the PHH3-assisted labels of the sutdy dataset: Despite imperfect alignment of HE- and PHH3-stained patches (lower row), the DI-FCOS model successfully detected MFs.

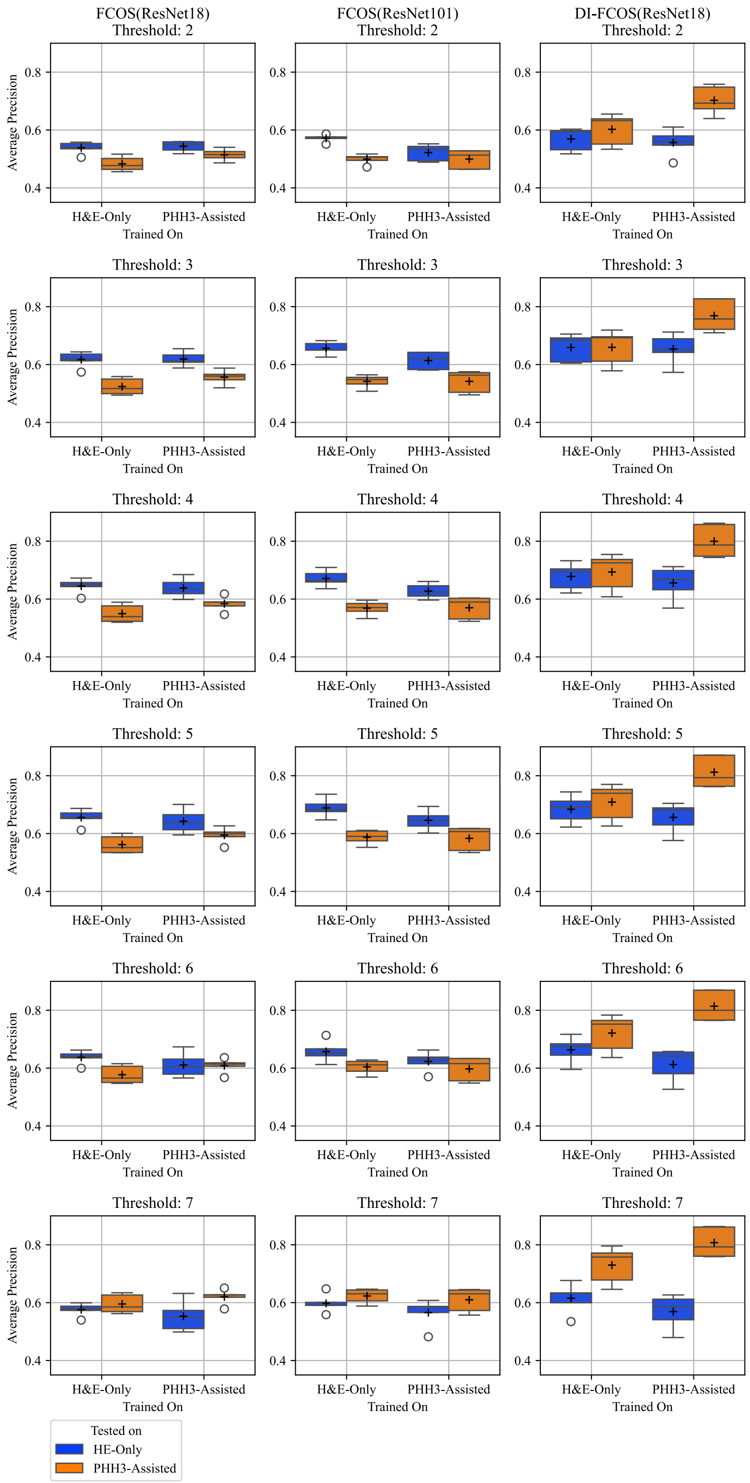

Fig. S3. Results of five-fold cross-validation of the FCOS and DI-FCOS models on the study dataset, as a function of the number of raters necessary to agree upon an object to be counted as a ground truth (GT) MF from two (top) to seven (bottom). For higher thresholds, the performance of the detector drops, as the number of GT MFs drops due to a high omission rate in the H&E annotations, while the detector performance is not altered significantly when using the PHH3-assisted annotation, as the omission rate is strongly reduced and rater agreement increased.

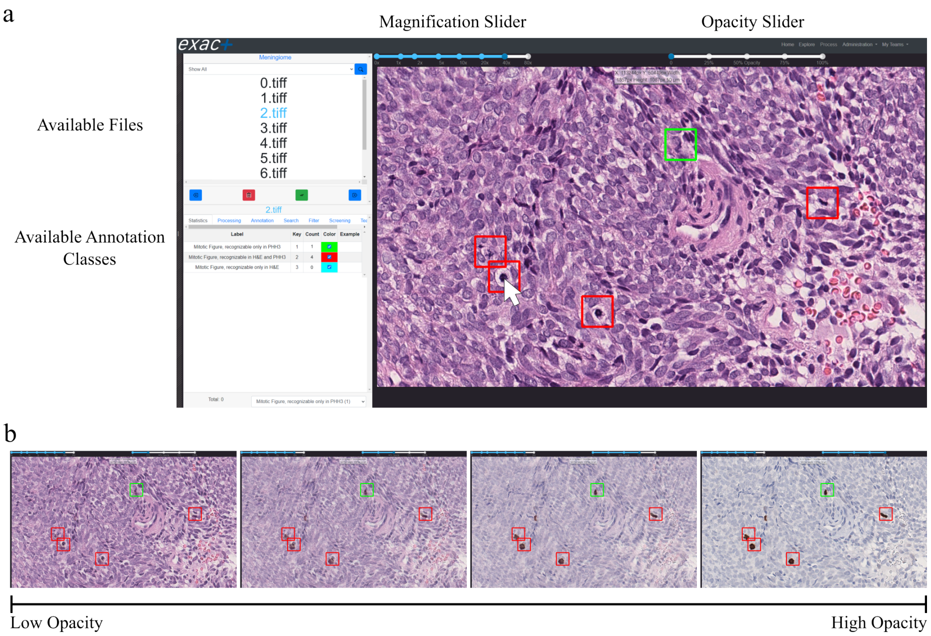

Fig. S4. a The experts were able to view the slides at different magnification levels. In the second phase of the study, they could also display the PHH3-stained slide as a transparent overlay over the H&E-stained slide. In the second phase of the study (P2), different annotation categories were available. Cells could be annotated by clicking on them. The bounding boxes had a default size of 50 by 50 pixels, but the size could be adjusted manually. b Since the registration was not always perfect, there was a slight displacement between the H&E- and the PHH3-stained slides. In this case, the experts were instructed to annotate the position of the respective cell in the H&E-stained slide.
